# *CORR* Insights®: Development and Internal Evaluation of EAST-BMS (East Asian Survival Tool for Bone Metastasis Surgery): A Multinational Machine-learning Survival Prediction Model for Patients Undergoing Surgery for Nonspinal Bone Metastasis in East Asia

**DOI:** 10.1097/CORR.0000000000004024

**Published:** 2026-06-16

**Authors:** Olivier Quinten Groot

**Affiliations:** 1 Resident, Department of Orthopaedic Surgery, University Medical Center Utrecht, Utrecht, the Netherlands

## Where Are We Now?

Accurately predicting life expectancy remains one of the central challenges in orthopaedic oncology. Patients with bone metastases typically have advanced systemic disease, and surgical decisions, including whether to operate, the choice of implant, and the extent of reconstruction, depend strongly on the patient’s life expectancy. Although overly aggressive interventions subject patients to unnecessary morbidity, underestimating survival can lead to needless and potentially preventable revisions in patients who survive over the longer term and who could have benefited from more durable reconstruction. Consequently, the choice between minimally invasive techniques and extensive surgery often hinges on accurate 90-day and 1-year survival thresholds [2, 9]. Several prognostic tools have been developed to aid both clinicians and patients in the decision-making process, but most of them were developed and validated in Western populations [12]. As cancer is a problem for patients all over the world, it may not be appropriate to assume that prognostic models validated only in North American and European populations apply equally well in other healthcare settings [15].

Prognostic models often perform well in the populations in which they are developed but may show decreased accuracy when applied to external cohorts [13]. Specific to bone metastases, differences in tumor biology, healthcare infrastructure, surgical indications, and systemic treatment strategies all contribute to this variability, raising concerns about the direct transferability of existing tools across regions and outcomes. For example, the management of spinal metastases differs between the United States and the Netherlands: In the United States, surgical interventions are more extensive and are often performed in patients with higher metastatic burden, whereas in the Netherlands, treatment more frequently emphasize less-invasive approaches. These differences reflect variation not only in treatment strategies, but also in surgical selection criteria and multidisciplinary practices, and they may translate into distinct survival patterns that underscore how regional context can influence prognostic performance [1].

In this issue of *Clinical Orthopaedics and Related Research*®, Yang et al. [17] address the limited generalizability of existing prognostic models by developing the East Asian Survival Tool for Bone Metastasis Surgery (EAST-BMS), a machine learning–based model derived from a multinational East Asian cohort. They showed that a regionally trained model can achieve modest but consistent improvements in calibration and clinical utility while reducing discrimination compared with an established Western model. Beyond performance metrics, their work provides an important conceptual advance: It demonstrates that tailoring prognostic tools to regional patient populations and practice patterns can enhance their clinical relevance. For surgeons, this reinforces that prognostic models are not universally applicable and thus should be selected with particular attention paid to the population in which they were developed. In practice, regionally validated tools should be preferred, and when using models developed elsewhere, predictions should be interpreted cautiously and within the context of clinical judgment.

## Where Do We Need To Go?

Although this study advances region-specific survival prediction, several important avenues remain for future research. First, as oncology continues to evolve, future models should incorporate tumor-specific and treatment-related variables to ensure that they reflect current survival patterns rather than historical ones [5]. Second, prognostic models should expand beyond survival alone to include other clinically relevant outcomes, such as perioperative complications, functional recovery, discharge location, and (perhaps most importantly) quality of life (QoL), which are all important for surgical decision-making. Finally, the implementation gap must be addressed; analyses must be conducted on how these tools actually alter surgeon behavior and the overall shared decision-making processes in the clinic. Only by demonstrating improved patient-centered outcomes in real-world settings will these tools see widespread adoption.

## How Do We Get There?

Moving from promising models to reliable, real-world clinical implementation requires a transition away from static datasets to seamless, automated data collection. Achieving this will call for standardized data fields across electronic health record systems so that oncologic, surgical, and outcomes data can be captured uniformly across institutions. In parallel, rather than relying on manual entry of structured tabular data such as demographics, laboratory values, and tumor characteristics, future research should leverage automated extraction from electronic health records to capture granular, tumor-specific variables such as histologic subtype, hormone receptor expression profile, mutation status, and response to novel treatment strategies [8]. This shift is not merely about increasing data volume, but about ensuring temporal relevance. By using “living” datasets that update alongside evolving oncology care, we can build dynamic models that remain aligned with current practice. This approach helps account for “concept drift,” the phenomenon where a model’s accuracy declines over time because the underlying patient population or treatment standards have changed [3, 7]. Such an approach ensures that prognostic tools remain calibrated to the contemporary therapeutic landscape rather than becoming outdated relics of the era in which they were first validated [5, 16].

Also, survival is only a piece of the shared decision-making puzzle. Expanding prognostic models to capture outcomes beyond survival is therefore essential, particularly for endpoints such as complications, functional recovery, and QoL. Although retrospective cohorts can provide insight into some postoperative outcomes, capturing QoL requires more structured and forward-looking data collection approaches. Unfortunately, to my knowledge, no validated prognostic models for QoL currently exist for patients with long-bone metastases [11]. In my opinion, QoL should be prioritized regarding outcomes for these patients as they navigate the final phase of life. Recent work by Kuijten et al. [10] in the field of spinal metastases has demonstrated that such modeling is indeed possible through prospective registries. Embedding these standardized, patient-centered measures into routine care would provide the foundation for models that more meaningfully inform real-world surgical decision-making.

Finally, demonstrating clinical usefulness requires moving beyond traditional performance metrics such as discrimination and calibration [14]. Future research should focus on prospective, head-to-head comparisons of artificial intelligence (AI)–aided predictions versus clinician judgment alone, evaluating whether these prognostic tools meaningfully improve decision-making and patient outcomes [18]. In an ideal scenario, this would be supported by integrated registries that bring together the aforementioned priorities, combining high-quality, automatically extracted data with continuous model updating and incorporating patient-centered outcomes such as QoL. Such platforms would allow for real-world testing of these tools within clinical workflows. Given that these steps entail substantial costs, economic evaluations alongside clinical utility studies are essential to determine whether improved predictive accuracy translates into meaningful cost-effectiveness and added value in practice [6]. Ultimately, we must aim for a future in which data-driven models and clinician expertise are no longer separate or adversarial but augment one another to the benefit of the patient.

## Read This Next

- This prospective series provides real-world evidence that AI-assisted predictions may enhance clinical judgment rather than replace it [18].
- This article offers a clear, practical framework for evaluating clinical prediction models [4].
